# Sit bath systems: A new source of *Legionella* infection

**DOI:** 10.1371/journal.pone.0241756

**Published:** 2020-11-04

**Authors:** Luna Girolamini, Marta Mazzotta, Jessica Lizzadro, Maria Rosaria Pascale, Ada Dormi, Silvano Salaris, Sandra Cristino

**Affiliations:** 1 Department of Biological, Geological, and Environmental Sciences, University of Bologna, Bologna (BO), Italy; 2 Department of Medical and Surgical Science, University of Bologna, Bologna (BO), Italy; University of Hong Kong, HONG KONG

## Abstract

Sit Bath Systems (SBSs) are the most common hygiene method for patients who are not self-sufficient. Therefore, the water quality of SBSs in the nosocomial environment plays a fundamental role in controlling infections for both patients and health-care workers. A long-term study on *Legionella* and *Pseudomonas aeruginosa (P*. *aeruginosa*) contamination was performed in SBSs (n = 20) of six Health Care Facilities (HCFs). A total of 254 water samples were analyzed following ISO procedures. The samples were positive for *P*. *aeruginosa* (46.85%) and *Legionella* (53.54%), respectively, both over the directive limits. *Legionella* isolates were identified as: *Legionella pneumophila* (*L*. *pneumophila*) serogroups 1, 3, and 6 and *Legionella* non-*pneumophila* species (*L*. *anisa*, *L*. *londiniensis*, *L*. *rubrilucens*, and *L*. *nagelii*). Moreover, the contamination found was studied with respect to median temperature measured (42 °C), from which two groups (A and B) could be distinguished. *P*. *aeruginosa* was found in both groups (100% of SBSs), while a higher percentage of *Legionella* positive samples was found in group A (75% of SBSs), compared to group B (50% of SBSs), showing how *Legionella* control could be carried out by using temperatures above 42 °C. An analysis of SBS water pipelines, maintenance, and disinfection treatments indicates SBSs as a new source of infection risk for both patients and health-care workers.

## Introduction

Nosocomial infections represent a significant public health issue, having potential impacts on the frequency and severity of disease, control of infection spread, and economic and social costs [1].

In Health Care Facilities (HCFs), the transmission of pathogens is facilitated by different exogenous and endogenous sources, such as patients, staff, surfaces, procedures, and devices that may constitute ecological bacteria niches [2]. In these environments, bacteria can persist for days or even months, colonizing surfaces, patients, or the hands of healthcare workers transiently, operating as vectors for bacteria transmission [3,4].

Opportunistic Premise Plumbing Pathogens (OPPPs) are a group of ubiquitous bacteria which are present in soil and are “regular” inhabitants of drinking water distribution systems. These bacteria mainly cause lung diseases but can also affect the skin and lead to other hospital-related infections, such as bedsores, superficial infections, and so on [5].

*P*. *aeruginosa* and *Legionella* are Gram-negative bacteria belonging to the OPPP group which represent sanitary risks, according to epidemiological data [6,7], and which are the main components of biofilm, where they find sustenance and protection from biocides and bacterial competition [8]. Moreover, they are able to switch from sessile biofilm to a free-living planktonic state due to changes in environmental conditions such as pH, nutrition level, and water flow that induce abrasion of the biofilm aggregate, permitting bacterial dispersion. In free-living planktonic state, the risks of transmission by water contact or aerosolization increase [9,10].

*P*. *aeruginosa* is an opportunistic human pathogen which is often resistant to multiple antibiotics [11,12]. Its optimal growth temperature is Σ37 °C but it can survive between 22–45 °C [13,14]. This bacterium can cause a number of different infections, including wound infections (particularly in patients with bedsores) [15–17], acute chronic respiratory infections, and sepsis [18,19].

*Legionella* spp. is found in both natural and artificial aquatic environments [19]. It is a causative agent of “Legionnaire’s disease” (LD) and Pontiac Fever [20]. The range of temperature permitting its survival is 5.7–63.0 °C, with an optimum between 25.0–42.0 °C [21].

In HCFs, the complex water distribution systems, variability of building characteristics, and health care delivery goals, can cause changes in water demands, increasing the risk of colonization and biofilm formation.

The drinking water used for showering/bathing, hand hygiene, and sterile processing, among other uses in patient care, with subsequent changes in microbial and chemical characteristics, can serve as a risk for patients and health-care workers; therefore, the control of nosocomial infection must be linked to water quality monitoring [22].

During environmental surveillance for *Legionella* infection, according to Italian and Regional Guidelines [23,24], a high level of contamination has been found in Sit Bath Systems (SBSs).

SBSs represent the devices used for hygiene for non-self-sufficient or bedridden patients, ensuring their health, comfort, and wellness, and often are the only method used. SBSs require specific maintenance and disinfection procedures, generally provided in the manufacturer’s manual. If these procedures are not properly carried out, the risk of cross-contamination between surfaces, patients, or healthcare workers can increase. Moreover, the microbial contamination of SBSs also depends on the water supply characteristics, such as physical–chemical parameters (i.e., temperature and pH) and other device technologies (e.g., pipeline systems or thermostatic mixing valves).

The aim of this study is to assess, for the first time, the risk of SBSs in nosocomial infections by evaluating the level of contamination, pipeline water network technologies, and disinfection procedures applied. This enables estimation of the impact of SBSs on the transmission of water-related pathogens such as *Legionella* and *P*. *aeruginosa* during a large-scale period of observation. There exist no specific references or guidelines identifying SBSs as critical points for the prevention of nosocomial infection, which may lead to underestimation of their risk.

## Materials and methods

### Health Care Facilities (HCFs)

Over a period of nine years (from 2010 to 2018), 20 SBSs located in six HCFs were monitored in order to assess the presence of *Legionella* and *P*. *aeruginosa* in the hot-water distribution system. All HCFs had adopted a risk assessment plan in accordance with Italian Regional Guidelines [23,24]. The Health Director of HCFs involved in the study authorized the collection of water samples. The laboratory, as a part of the University of Bologna, had the permission to carry out the *Legionella* environmental monitoring, preserving Hospitals’ anonymity. Our study did not involve endangered or protected species.

The main characteristics of the six HCFs (alphabetically listed), in relation to their year of construction, number of floors, number of rooms, beds in the structure, and the number of SBSs, are presented in Table 1.

**Table 1:** Characteristics of health care facilities.

ID Structure	Year of construction	Numbers of floors	Number of rooms	Numbers of beds	Numbers of SBSs
**A**	2002	4	53	97	4
**B**	1924	6	35	66	3
**C**	2011	3	78	120	4
**D**	1989	4	38	100	3
**E**	1997	2	10	21	2
**F**	1962	4	71	100	4

The hot-water systems were treated with disinfectant containing hydrogen peroxide and silver salts (H_2_O_2_/Ag^+^), injected into the hot-water return line points by electronic pumps. This was dosed proportionally to the water supply (at a concentration of around 50 mg/L), allowing for a residue at the outlets of 10–20 mg/L, following the manufacturer’s instructions.

### Sit bath system characteristics

Sit baths are employed for the assisted bathing and showering of patients in hospitals and HCFs. It is compulsory to use a sit bath only if caregivers are appropriately trained with adequate knowledge of the care environment, its common practices, and procedures, as well as in accordance with the guidelines provided by the manufacturer, which establish regular assessment routines:

- Patient weight should be less than 160 kg.
- The patient must have mental capacity and must have the ability to move (even if limited), to ensure a safe position during the bathing procedure. If this is not possible, the patient should use alternative bathroom equipment/systems.

**Fig 1:**
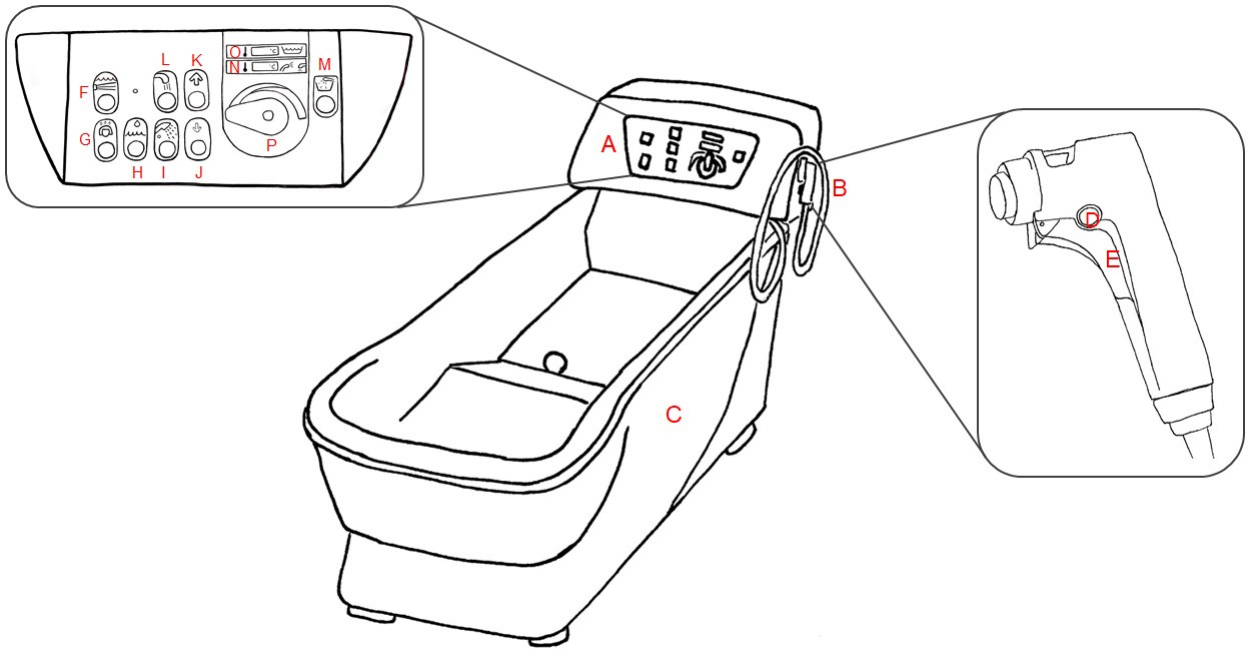
Representation of sit bath system: A) Control panel; B) Shower head; C) Bathtub; D) Lock button; and E) Trigger.

A representative scheme of the SBS studied is shown in Fig 1; the main components are: bathtub, control panel, and pipe with shower head.

In the control panel (Fig 1A), there are two displays: one showing the water temperature inside of the bathtub (Fig 1O), and another indicating the water temperature in the flow through the shower head (Fig 1N). The regulation of temperature (cold/hot) is performed using a handle (Fig 1P); the control panel also provides other functions (e.g., start and stop hydromassage, erogation of shampoo and body wash, erogation of water from the shower head, lowering and raising the height of the bathtub, and so on).

**Fig 2:**
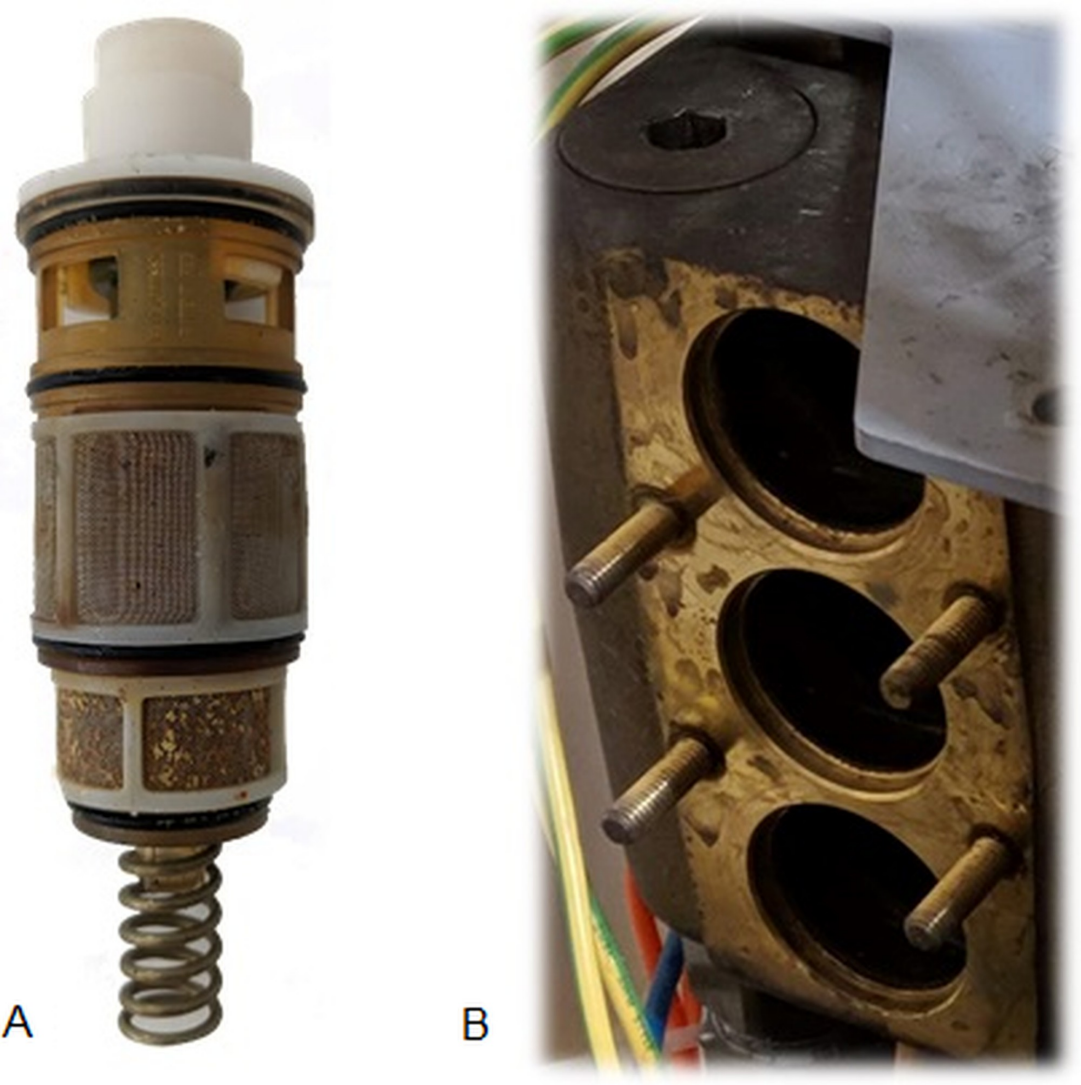
Representation of SBS mixer-cartridges (A) and slots of mixer-cartridges (B).

SBSs are supplied by municipal water transmitted through two pipelines (hot and cold), which pass into three mixer-cartridges which produce mixed water, where the temperature is regulated by thermostatic valves (see Fig 2).

SBSs are equipped with electric scalding protection, designed to increase patient safety. This function is used during SBS filling, showering, and cleaning procedures. The highest temperature that an SBS can reach is 43 ± 3°C, defined as the temperature to avoid scalding risks to users [25]. The scald temperature can vary individually, especially with regard to their age. The scalding protection is activated when the water temperature gets too high. If the bathtub water reaches 45 °C or above, the electronic scalding protection shuts off the water after 10 seconds, while both LEDs on the Disinfection/Auto clean button and the patient shower button flash for a short time. Therefore, in order to prevent scalding (if scalding protection is activated), the patient must be removed from the bath immediately, in a safe manner. After that, the healthcare worker can reset the scalding protection.

### Cleaning and disinfection procedures

Disinfection has the aim of minimizing cross-contamination between patients and skin residues released in SBSs. The disinfection process can only be started when all other SBS functions are turned off. The schedule of preventive functional maintenance procedures suggested by the manufacturer (Table 2) must be carried out with the correct frequency by qualified personnel after adequate training. The disinfectant suggested by the manufacturer was based on a benzalkonium chloride (BAC) product, as indicated for only the surfaces and showerheads of SBSs. The cleaning procedures and maintenance activities were recorded in a dedicated register and validated monthly by HCF staff.

**Table 2:** Preventive functional maintenance schedule (from the manufacturer’s manual).

Caregiver Obligations Action/Check	Between patients	Every Day	Every Week	Every Month	Every Year
**Clean and disinfect (BAC disinfectant to mix with water at 25 °C)**	X				
**Check liquid levels**		X			
**Visually check all exposed parts**			X		
**Visually check hoses, pipes, and connections**			X		
**Perform functionality test**			X		
**Hydrosound system functionality test**			X		
**Check and clean shower head**				X	
**Check and clean filters located at input of municipal water**				X	
**Exercise the thermostatic valve**				X	
**Yearly functional checks by manufacturer personnel**					X
**Check electrical installation (to be performed by authorized electrician)**	Intervals of checking to perform according to HCF requirements				

### Collection of hot water samples

Following the *Legionella* risk assessment plan for HCFs and according to the Guidelines [23,24], the SBSs were monitored two times per year to analyse water quality. From 2010 to 2018, 20 SBSs were sampled for a total of 256 samples. Following the *Legionella* Guidelines, the level of risk must consider the concentration of bacteria and the percentage of positive samples in the total number of samples analysed.

The sampling was then repeated when the results were above the Legislation limits.

For each SBS, 2 liters of hot water were collected from the showerhead (erogation point of water, point B in Fig 1), according to the standardized procedures suggested by UNI EN ISO 19458:2006 [26] and Italian Guidelines [23]. The water was collected in the morning before the patient’s personal hygiene routine, in particular to test the quality in the main distribution system. Post-flushing sampling was applied (running water for about 1 minute) in sterile polytetrafluoroethylene (PTFE) bottles containing a sodium thiosulfate solution (20 mg/L). The bottles were maintained at 4 °C until analysis.

### Physical and chemical analyses

The physical and chemical parameters were measured during sampling, corresponding respectively to temperature and disinfectant residues of the water sampled from SBS showerheads. Temperature (°C) was measured by a conductivity meter coupled with a thermistor probe (Temp 6 basic for probe Pt100 RTD from –50 to +199 °C; Eutech Instruments Pte Ltd., Singapore). The residual H_2_O_2_ component of H_2_O_2_/Ag^+^ was measured (mg/L) on-site using a commercial kit. The kit used a colorimetric test based on peroxidase activity to transfer peroxide oxygen to an organic redox indicator; this produces a blue oxidation product. The H_2_O_2_ concentration was measured semi-quantitatively by visual comparison of the result seen on the reaction zone of the test strip with the fields on a colour scale, in a range of 1–200 mg/L.

### Microbiological analysis

Hot water samples were analysed for the presence of *P*. *aeruginosa* and *Legionella*.

Analysis of *P*. *aeruginosa* was performed using a standard membrane filter technique on *Pseudomonas*-selective agar (PSA, Biolife, Milan, Italy), according to UNI EN ISO 16266:2008 [27]. For each sample, 100 mL was filtered using a cellulose nitrate membrane filter with 0.45 μm pore size (Sartorius Stedim Biotech, Göttingen, Germany). The typical colonies of *P*. *aeruginosa* grew on selective media, showing green–blue fluorescence under Woods lamp (UV light at 365 nm). These colonies were also sub-cultured and subjected to biochemical identification. Typing was carried out using a BBL Crystal Enteric/Non-Fermenter ID kit (Becton Dickinson Systems, Cockeysville, MD, USA), according to the manufacturer’s instructions [28]; additionally, indole and oxidase reactions in isolates were tested. Data are expressed as mean concentration ± standard deviation (SD) of log_10_ (Log) colony forming units (cfu) per 100 mL of water (log_10_ cfu/100ml or Log cfu/100ml), according to directive limits [22].

*Legionella* was isolated by the culture method according to ISO 11731:2017 [29]. For the enumeration of *Legionella*, different aliquots of samples (untreated, filtered, and treated by heat and acid) were directly plated onto the *Legionella*-selective medium Glycine-Polymyxin B-Vancomycin-Cycloheximide (GVPC; Thermo Fisher Scientific, Oxoid, Ltd., Basingstoke, U.K.). All plates were incubated aerobically at 35 ± 2 °C and 2.5% CO_2_ for up to 15 days.

Colonies with morphology associated to *Legionella* genus were enumerated and sub-cultured on buffered charcoal yeast extract (BCYE) agar, both with (BCYE cys+) and without (BCYE cys-) L-cysteine (Thermo Fisher Scientific, Oxoid, Ltd., Basingstoke, U.K.). Isolates that grew on BCYE cys+ but failed to grow on BCYE cys- were verified serologically by an agglutination test (*Legionella* latex test kit; Thermo Fisher Scientific, Oxoid, Ltd. Basingstoke, UK). This test permitted distinguishing between *L*. *pneumophila* SG1, SG 2–14, and seven other *Legionella* non-*pneumophila* species which are involved in human disease. The *L*. *pneumophila* colony which were SG 2–14 positive were then tested for single serogroups by specific polyclonal latex reagents (Biolife, Milan, Italy).

*Legionella* concentrations are expressed as mean concentration ± SD of log_10_ (Log) colony forming units (cfu) per liter of water (Log cfu/L), including all samples collected/analysed (log_10_ cfu/L (Log cfu/L)) [23,24]. According to ISO 11731:2017, negative results were indicated as those below the detection limit of the technique which, for 2-liter samples, corresponded to <50 cfu/L (1.7 Log cfu/L), while the directive risk level was fixed as >100 cfu/L (>2 Log cfu/L) [23,29].

Furthermore, some swab samples were collected from the inside of the pipe after removing the showerhead and inside the slots of the SBS mixer-cartridges (Fig 2B), to analyse aggregates biofilm.

The swab samples were resuspended in 20 mL of Ringer’s solution (Thermo Fisher Scientific, Oxoid, Ltd., Basingstoke, U.K.) and processed for *P*. *aeruginosa* and *Legionella* cultures, according to UNI EN ISO 16266:2008 and ISO 11731:2017, respectively [27,29]. Cultural, morphological, and biochemical characterizations of isolates were performed as described above.

The results of swab samples, according to the Guidelines, were qualitative: Absence or presence.

### *mip* gene sequencing

The isolates typed as non-*pneumophila Legionella* species were genotyped by *mip* gene sequencing, according to method described by Ratcliff et al. [30].

In detail, DNA extraction was carried out using the InstaGene Purification Matrix (Bio-Rad, Hercules, CA) and DNA concentrations were determined using the Qubit fluorometer (Thermo Fisher Scientific, Paisley, U.K.). The *mip*-gene amplification was carried out in a 50 μL reaction containing DreamTaq Green PCR Master Mix 2× (ThermoFisher Diagnostic Basingstoke, UK) and 40 picomoles of each primer. Furthermore, 100 ng of DNA extracted from the presumptive colonies was added as template. The M13 forward and reverse primers (*mip*-595R-M13R caggaaacagctatgaccCATATGCAAGACCTGAGGGAAC and *mip*-74F-M13F tgtaaaacgacggccagtGCTGCAACCGATGCCAC) were used to obtain complete coverage of the sequenced region of interest [31].

PCR products were visualized by electrophoresis on 2% agarose gel and stained with ethidium bromide. Following purification, DNA was sequenced using BigDye Chemistry and analysed using an ABI PRISM 3100 Genetic Analyzer (Applied Biosystems, Foster City, CA).

Raw sequencing data were assembled using the CLC Main Workbench 7.6.4 software. The sequences were compared to sequences deposited in the *Legionella mip*-gene sequence database using a similarity analysis tool (http://bioinformatics.phe.org.uk/cgi-bin/Legionella/mip/mip_id.cgi). Species-level identification was carried out on the basis of ≥98% similarity to a sequence in the database [32].

### *Legionella* disease surveillance

In HCFs, all patients with suspected pulmonary signs of pneumonia underwent urine antigen testing and other diagnostic tests (e.g., chest X-ray) to confirm diagnosis, according to Italian Guidelines and ESGLI criteria [23,33].

### Statistical analysis

The level of contamination for *P*. *aeruginosa* and *Legionella* data were converted into log_10_ cfu/100 mL (Log cfu/100 mL) and log_10_ cfu/L (Log cfu/L), in order to normalize non-normal distributions for the correlation analysis.

Statistical analyses were performed using the SPSS software for Windows version 25 (IBM SPSS, Inc., Chicago, IL, USA). The normality of variables was assessed using the Shapiro–Wilk test. The Mann–Whitney test and the bivariate correlation procedure was performed for non-parametric variables indicated by the Spearman–Rho coefficient (r_s_). The t-test was performed to compare bacterial contamination over and below 50 °C, as analysed in SBSs 1, 3, and 19 (Table 5). Significance of all statistical tests was set by *p* value (*p*) ≤ 0.05.

## Results

During the study, 254 samples were collected and analysed from 20 SBSs. Data relating to the mean *P*. *aeruginosa* and *Legionella* concentrations are presented in Table 3, including the mean temperature and H_2_O_2_ residue concentrations measured in each SBS for each year of monitoring. The results are expressed either as mean ± SD or median ± Interquartile Range (IQR).

**Table 3:** Overview of data collected at the six HCFs during the study (mean of temperature, H_2_O_2_, *P*. *aeruginosa*, and *Legionella* concentration/year of monitoring).

ID structure	Year of monitoring	Temperature (°C)			H_2_O_2_ (mg/L)	*P*. *aeruginosa* concentration (Log cfu/100mL)			*Legionella spp*. Concentration (Log cfu/L)		
		Mean	± SD	Mean Minimum (Min)–Maximum (Max)	Mean	Mean	± SD	Mean Min–Max	Mean	± SD	Mean Min–Max
**A**	2010	43.00	2.00	41.80–47.53	10–20	2.53	1.74	0–2.53	2.35	0.92	1.20–2.50
	2011	47.53	3.83			1.32	0.93		1.20		
	2012	44.13	1.44			1.31	1.17		1.50	0.59	
	2013	43.78	1.45			2.01	1.33		2.15	1.10	
	2014	43.98	1.76			1.45	0.81		1.20		
	2015	45.98	3.88			1.21	1.19		1.43	0.66	
	2016	42.78	6.77			1.75	1.07		2.22	0.97	
	2017	42.65	1.09			2.47	0.51		2.50	1.00	
	2018	41.80	2.23			2.14	0.45		2.16	1.03	
**B**	2010	38.47	2.20	29.67–39.56	10–20	1.05	1.20	0–1.05	3.43	1.32	1.20–3.43
	2011	34.27	1.97			0.32	0.55		1.20		
	2012	29.67	4.65			0			2.61	0.71	
	2013	31.30	2.35			0.10	0.17		2.44	0.13	
	2014	39.43	1.98			0			2.36	1.10	
	2015	35.93	2.48			0			3.30	0.35	
	2016	38.70				0			1.20		
	2017	39.56	4.11			0			1.80	0.66	
	2018	39.00	4.84			0.23	0.62		2.21	0.78	
**C**	2010			41.40–42.80	10–20			0–0.85			1.20–2.24
	2011	41.40	0.79			0.73	0.63		1.20		
	2012	41.95	0.39			0.50	0.63		1.20		
	2013	41.60	0.14			0.70	0.99		2.24	1.47	
	2014	42.60	0.28			0.53	0.60		1.20		
	2015	42.10	0.42			0.85	1.20		1.20		
	2016	42.80				0			1.20		
	2017	42.20	1.36			0.21	0.29		1.20		
	2018	41.90	0.75			0.17	0.38		1.20		
**D**	2010	42.33	1.53	38.43–45.0	10–20	1.18	1.55	0–0.83	4.41	1.24	1.63–4.41
	2011	42.00	5.66			0			2.83	0.12	
	2012	45.00	3.46			0.49	0.85		4.19	0.52	
	2013	43.53	2.25			0.65	1.13		3.99	0.17	
	2014	38.43	6.08			0.77	1.33		2.8	1.39	
	2015	43.20	2.55			0.83	1.14		1.96	1.04	
	2016	42.73	1.17			0			2.25	0.13	
	2017	41.82	1.98			0			1.63	0.69	
	2018	41.40	2.87			0.07	0.18		1.66	0.43	
**E**	2010	39.75	2.82	39.65–44.90	10–20	0.55	0.82	0–2.03	3.76	0.97	1.20–3.80
	2011	38.50	2.38			0.44	0.70		3.80	0.77	
	2012										
	2013										
	2014	42.03	3.39			2.03	0.96		3.68	1.77	
	2015	44.43	2.62			0			2.67	0.99	
	2016	44.90	3.54			0			1.70		
	2017	39.65	3.32			0.35	0.49		1.20		
	2018	39.80	3.41			0.10	0.17		3.12	0.20	
**F**	2010	41.57	2.12	40.83–45.34	10–20	1.32	1.01	0–1.32	3.74	1.28	1.32–3.74
	2011	40.85	1.31			1.19	1.07		3.39	1.48	
	2012	42.80	1.44			0.31	0.62		2.66	1.19	
	2013	40.83	2.15			0.1	0.14		2.85	0.78	
	2014	42.95	4.01			0.58	0.68		1.32	0.25	
	2015	44.15	3.47			1.00	1.12		1.95	1.50	
	2016	45.34	4.32			0.13	0.43		1.68	0.83	
	2017	43.55	1.30			0.10	0.19		1.47	0.59	
	2018	45.24	2.97			0.03	0.10		1.62	0.68	

All SBSs (100%, 20/20) were contaminated by *P*. *aeruginosa*. Positive samples were 46.85% (119/254), with concentration between 0.1 and 3.98 Log cfu/100 mL, all of them over the directive risk level fixed as 0 cfu/100mL [22,23]. Typing confirmed the presence of *P*. *aeruginosa* species in all samples.

The *Legionella* results showed that 85% of SBSs (17/20) were contaminated. Positive samples were 53.54% (136/254), with contamination values between 1.70–6.03 Log cfu/L. Negative samples, according to ISO 11731:2017 and Italian Guidelines [23,29], were considered those with concentration below the detection limit of the technique, corresponding to <1.70 Log cfu/L.

Regarding the directive risk level (fixed to >2 Log cfu/L), 60% of SBSs (12/20) showed contamination over this level. Serotyping indicated that the main *Legionella* isolates belonged to *L*. *pneumophila* (serogroups 1, 3, and 6) and non-*L*. *pneumophila* species. The results of *mip*-gene sequencing identified isolates of *L*. *anisa*, *L*. *londiniensis*, *L*. *rubrilucens*, and *L*. *nagelii*, matching reference strains by ≥98%.

**Fig 3:**
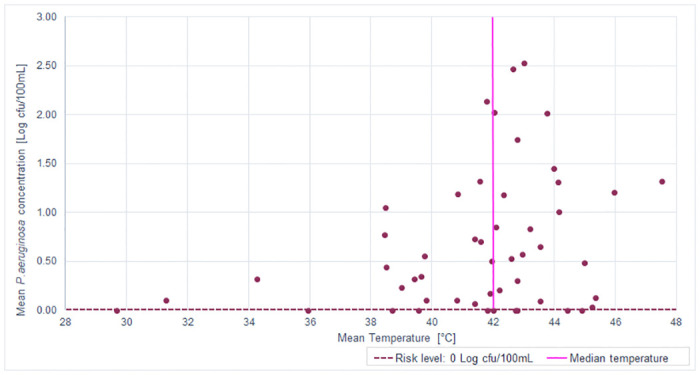
Distribution of mean *P*. *aeruginosa* concentration in relation to SBS median temperature.

**Fig 4:**
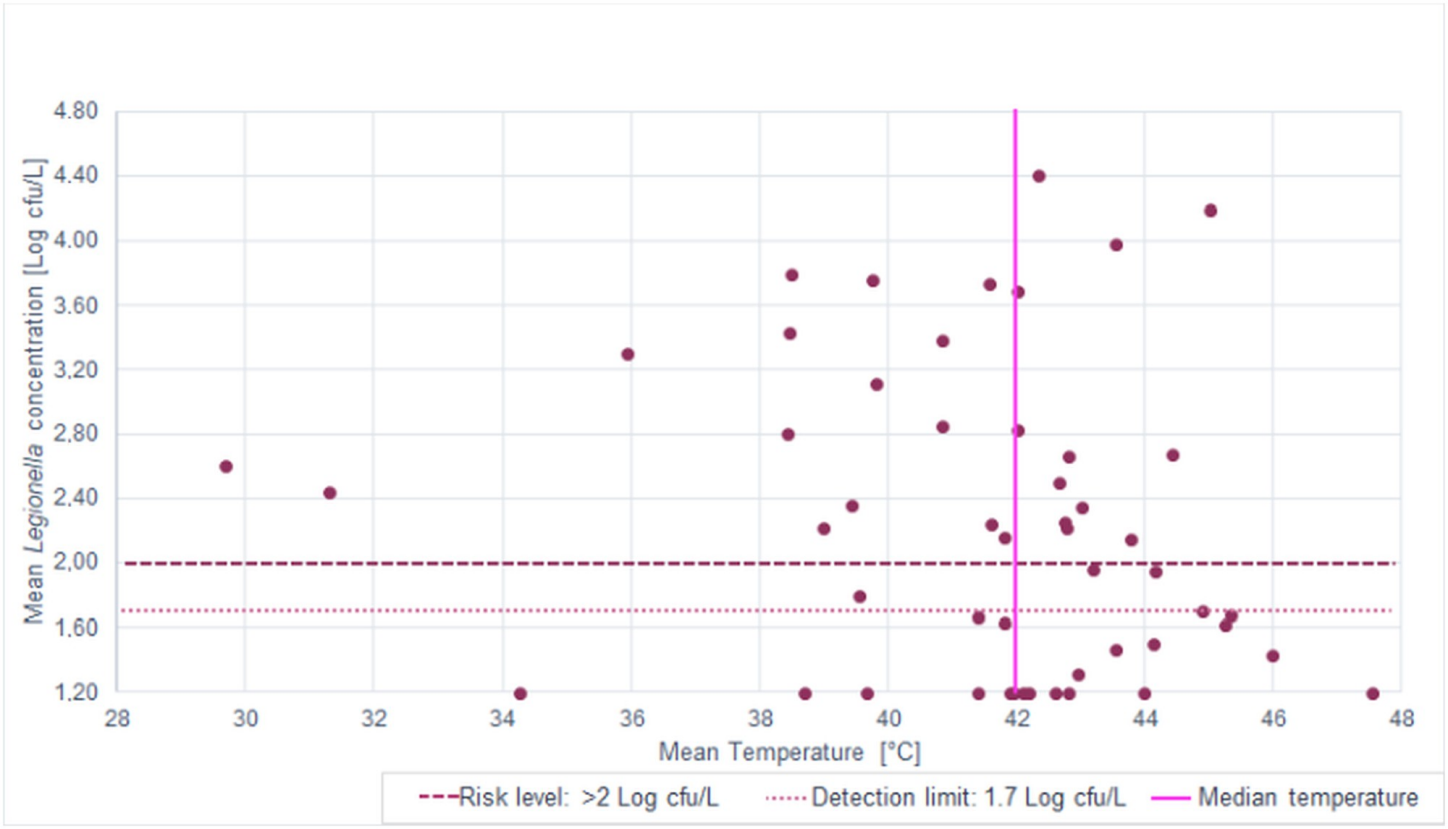
Distribution of mean *Legionella* concentration in relation to SBS median temperature.

The SBSs working temperature range was between 29.67–47.53 °C (Table 3), with median of 42.0 ± 3.38 °C. Correlations between temperature and microbiological parameters analysed are shown in Figs 3 and 4, respectively, for *P*. *aeruginosa* and *Legionella*.

These figures demonstrate how the main positive samples—67.50% (27/40) for *P*. *aeruginosa* and 64.52% (20/31) for *Legionella*—were inside the range of temperature of 40.0–45.0 °C. This range of temperature permits optimal growth for these bacteria, which has been previously documented [13,14,21].

The correlation between the range of temperature measured in SBSs (29.67–47.53) and microbiological parameters analysed was performed using the Spearman–Rho coefficient (r_s_). This demonstrates how the correlation between *P*. *aeruginosa* concentration and temperature was positive: the increase of temperature values caused a significant increase of *P*. *aeruginosa* concentration (r_s_ = 0.260; *p* = 0.065). In contrast, we observed a negative correlation for *Legionella* concentration (r_s_ = -0.220; *p* = 0.121): the increase of temperature values caused a decrease in *Legionella* concentration. The analysis of results with respect to the median temperature found (42 ± 3.38 °C) permitted us to distinguish two main groups between SBSs:

- Group A, represented by 40% (8/20) of SBSs, with T ≤ 42.0 °C; and
- Group B, represented by 60% (12/20) of SBSs, with T > 42.0 °C.

The contamination levels for *P*. *aeruginosa* and *Legionella* in these two groups are given in Table 4.

**Table 4:** *P*. *aeruginosa* and *Legionella* contamination levels in SBSs.

Micro-organisms	Group	Temperature (°C)	SBSs contaminated over risk level (%)	Mean ± SD contamination level	Minimum contamination level (Min)	Maximum contamination level (Max)	Mann–Whitney test *p* value
*P*. *aeruginosa* (Log cfu/100mL)	A (25/51)	≤42.0	100%	0.43 ± 0.53	0	2.14	0.036*
	B (26/51)	>42.0	100%	0.88 ± 0.79	0	2.53	
*Legionella* (Log cfu/L)	A (25/51)	≤42.0	75%	2.37 ± 0.91	1.70	3.80	0.275
	B (26/51)	>42.0	50%	2.11 ± 0.98	1.70	4.41	

*Values are statistically significant at *p* < 0.05.

**Fig 5:**
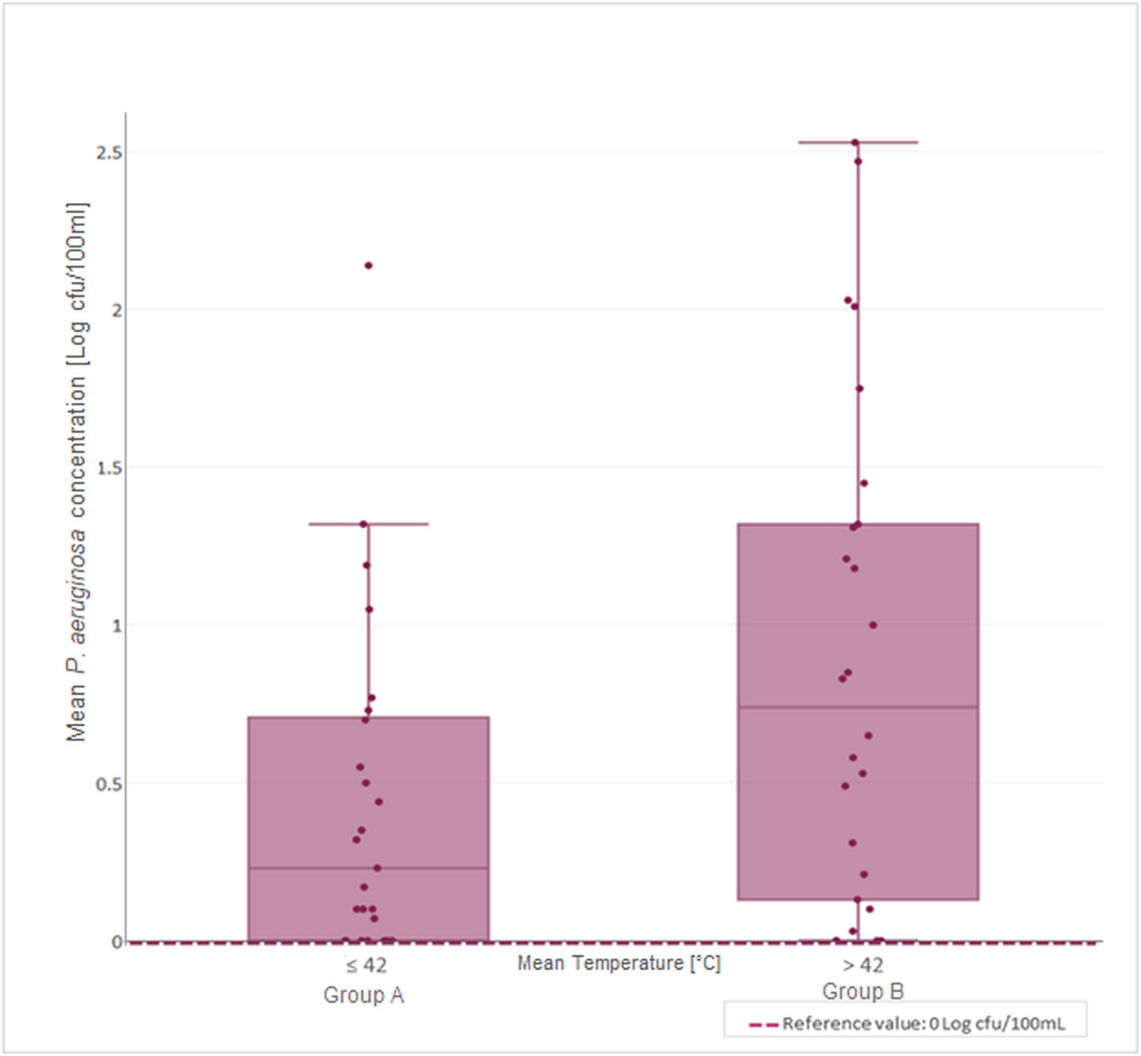
*P*. *aeruginosa* mean concentration distribution in the two groups (A vs. B) with respect to the median temperature.

**Fig 6:**
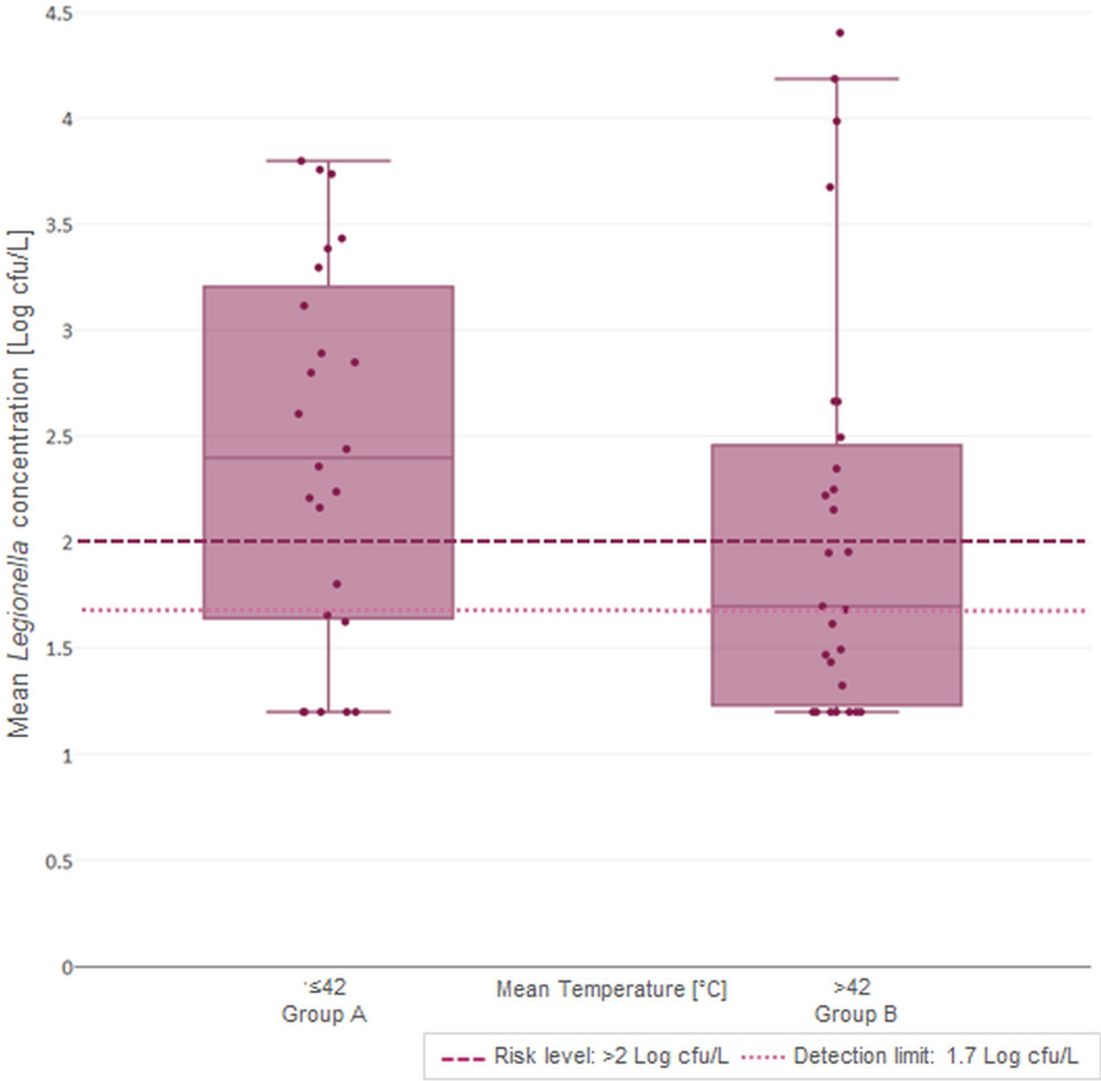
*Legionella* mean concentration distribution in the two groups (A vs. B) with respect to the median temperature.

In Figs 5 and 6, we show graphical representations of the contamination reported for *P*. *aeruginosa* and *Legionella*, respectively, compared to the median temperature found (42.0 ± 3.38 °C).

As reported in the figures and according to the Spearman–Rho coefficient, higher temperatures caused a significant increase in the mean *P*. *aeruginosa* concentration from 0.43 to 0.88 Log cfu/100mL, a percentage increase of 103.94% (*p* value = 0.036). By contrast, we found a decrease from 2.42 to 2.08 Log cfu/L of mean *Legionella* contamination, corresponding to a percentage decrease of 14.31% (*p* value = 0.275).

During the monitoring performed in the course of the study, for the years 2011, 2015, 2016, and 2018, the HCFs A and F, with the SBSs 1, 3, and 19, showed some samples (n = 6) with temperature values above 50 °C.

The comparison of *Legionella* and *P*. *aeruginosa* mean concentrations recorded in these samples were compared with the values found in the samples below 50 °C, which showed a statistically significant difference for both parameters (*p* value = 0.00001).

The results obtained from these SBSs are given in Table 5.

**Table 5:** *Legionella* and *P*. *aeruginosa* comparison in SBSs with temperature above 50 °C.

ID HCF	ID SBSs	Range of temperature (°C)	Mean temperature	*Mean P*. *aeruginosa* (Log cfu/100mL)	*Mean Legionella* (Log cfu/L)	t-student test *p* value
A, F	1, 3, 19	<50	43.16	1.39	2.16	0.00001*
		>50	51.45	0	1.2	

*Values are statistically significant at *p*<0.05.

Furthermore, swabs performed inside SBS showerhead pipes and in slots containing mixer cartridges during the study showed the presence of biofilm aggregates containing isolates mainly belonging to *P*. *aeruginosa* and *L*. *anisa*.

### LD surveillance

Concerning the active surveillance of legionellosis performed in HCFs during our study, two cases of suspected Legionellosis, as diagnosed by positive urinary test and positive pulmonary X-ray, were reported. In both cases, the epidemiological investigations found positive samples in SBSs and in other points of HCFs; however, the clinical isolates from bronchoalveolar lavage were negative, as the culture of *Legionella* from this material is difficult due to presence of interference microflora which, other than during the fast treatment of patients with broad-spectrum antibiotics, can interfere with culture results. Therefore, in the absence of culture from clinical samples, detection and linking with source of infection was not possible [29,33].

## Discussion

Nosocomial infection control is based on the identification of new sources of infections, infectious doses, and virulence, other than their transmission potential and host susceptibility. The most critical environment is represented by hospitals, where new procedures, technologies, and devices are continually introduced to improve the assistance, care, and hygiene of patients [34].

The control of nosocomial infections is aimed at maintaining health and safety, for patients as well as for health-care workers. In fact, the latter, without adequate personal protective equipment, could come into contact with micro-organisms during the assistance of patients or when using devices (e.g., urinary catheters, dental practices, respiratory therapy equipment, and nasogastric feeding) that are often cleaned with unsterilized tap water or removed incorrectly [9]. One of the main sources of infection in HCFs is represented by (hot and cold) water supplies for the hygiene of patients and healthcare worker activities.

However, the association between *Legionella* infection and work activities has been poorly documented and is difficult to evaluate, compared to other occupational diseases [35,36].

Epidemiological data and infection control experience have highlighted the importance of water management, in terms of building characteristics, plumbing systems, and the training of clinical staff, with respect to water-related infections [9,37,38].

This study represents the first long-term (nine years) and large-scale (six HCFs) monitoring of *P*. *aeruginosa* and *Legionella* contamination in SBSs, demonstrating their impact on the transmission of water-related pathogens. The analysis of *P*. *aeruginosa* was linked to the role of this bacterium in the SBS environment, where elderly and immunocompromised patients, who often have damaged skin or bed sores, come into contact with water or surfaces which may be contaminated.

Contamination by these bacteria was investigated by sampling the water supply in the showerhead of SBSs, in order to reproduce the water environment that comes in contact to patients and healthcare workers, according to previous findings which have demonstrated that exposure to showers represents the greatest risk of acquiring infection for immunocompromised and elderly people residing in HCFs [33,39].

The high rate of SBS contamination by *P*. *aeruginosa* (100%) and *Legionella* (85%), often over the regulation limit for both parameters [22,23], suggests that SBSs represent critical devices which are often underestimated, especially in long-term care facilities [6,11,12,16,17]. Moreover, our results showed that SBSs represent a widely contaminated environment for *Legionella* species; indeed, we isolated species frequently associated with human disease, such as *L*. *pneumophila* and *L*. *anisa*, as well as species whose pathogenic impact has been less documented and studied, such as *L*. *londiniensis*, *L*. *rubrilucens*, and *L*. *nagelii*. Regarding Pseudomonadaceae, only *P*. *aeruginosa* species were found, which have been largely associated with hospital-acquired infections.

Moreover, the presence of both pathogens was correlated with warm temperatures, technologies used, and the maintenance protocols applied.

Regarding the temperature, the most positive samples for both parameters were found within 29.67–47.53 °C, with a median of 42.0 ± 3.38°C. This corresponded to optimum bacterial growth rates, which have been placed between 25–42°C for *Legionella* [21] and between 22–45 °C for *P*. *aeruginosa* [13,14]. In SBSs, the temperature showed a different impact on these micro-organisms, as demonstrated by the Spearman–Rho coefficient values: an increase of temperature caused a significant increase in *P*. *aeruginosa* concentrations (r_s_ = 0.260, *p* = 0.065), whereas we observed a decrease for *Legionella* (r_s_ = -0.220, *p* = 0.121). The (non-significant) *Legionella* data could be explained by the working SBS setting, which is strictly closed to the *Legionella* growth range; indeed, *Legionella* is able to survive in the presence of temperatures over 50 °C [13,14,21]. Moreover, its inactivation depends on the time of exposure to higher temperatures [40].

Additional evaluations were conducted, with respect to the median temperature (42.0 ± 3.38°C), from which we distinguished two groups of SBSs: A and B. This value corresponds also to the functional setting value suggested by the manufacturer (43 ± 2 °C), which assures the good performance of SBSs and, at the same time, avoids the risk of scalding patients and healthcare workers. In line with previous results, the concentration of *P*. *aeruginosa* showed a significance percentage of increase between groups, which could be explained by the positive effect of temperature in assuring an optimal environment for the growth of *P*. *aeruginosa*, as previously described [13,14]. This evidence was also supported by ISO 11731:2017 for *Legionella* culture techniques, which requires heat (50 °C for 30 minutes) and acid treatment on samples to inhibit the growth of *P*. *aeruginosa* or other competitors, avoiding false negative results [29].

By contrast, the evaluation of *Legionella* concentration between two SBSs groups showed a small percentage of concentration decrease (-14.31%) that, despite non-significant *p*-value, demonstrated how a short increase of temperature in SBSs was able to produce an effect on bacterial growth. This effect could promote H_2_O_2_/Ag^+^ disinfectant activation, in line with a previous study which required a hot temperature for its activation [41].

The contamination found was also studied by analysing the SBS technologies to produce hot temperatures. The SBS working temperature is based on a thermostatic valve, where mixer cartridges start by mixing the two water circuits—hot and cold—to obtain mixed water with a maximum working temperature of 42 ± 3 °C. The installation of thermostatic valves at outlets has been promoted by different local regulations [23], in order to allow a balance between hot water, scald injury, and infection prevention. As reported for other devices (e.g., sensor-activated faucets), thermostatic valves may enhance bacterial growth [42] by producing water temperatures conducive to *Legionella* growth (as well as for other pathogens), especially in outlets such as SBSs, with low disinfectant concentrations, lacking routine maintenance protocols, and only periodic assessment of water microbiological quality [9].

Starting from these considerations, the evaluation of SBS water pipelines and maintenance protocols, as suggested by the manufacturers and adopted by personal staff, was performed.

The analysis of SBS water pipelines systems showed a complex pipelines system made of plastic material that, due to its small diameter (1–2 cm maximum), was often full of biofilm formation inside the internal diameter. It is likely that biofilm growth is enhanced by the low pressure and inconsistent flushing of water outside the occasional use of SBSs (generally occurring only during the first part of morning). Such low pressure and flushing, along with the stagnation of water, may intensify *Legionella* colonization and proliferation, as well as increasing biofilm formation [33,43]. Moreover, no descriptions or suggestions have been made by the manufacturer with respect to SBS internal components, with specific reference to mechanically checking and replacing those internal components that are essential to preserving the functionality of the device and contributing to maintaining water quality (e.g., substitution or cleaning of mixer cartridges).

Concerning the ordinary disinfection procedures provided in the SBS manuals, BAC disinfectant is suggested for use only on SBS surfaces and showerheads. As previously documented, BAC has shown different activities on bacteria, with Gram-positive generally being more susceptible than Gram-negative bacteria [44]. Moreover, its activity depends on the surfactant concentration, bacterial concentration, the high temperature value, and length of exposure time [45]. Despite the regulation EN 14885:2018 [46] suggesting an active dosage at 0.5% v/v with an exposure time of up to 1 h for surfaces in health care settings, no information about concentration, contact time, and the schedule of timing have been reported in the manual.

Although the role of disinfection treatments—in particular, physical treatment (e.g., flushing, superheating) and chemical treatment (e.g., chlorination by chlorine dioxide, monochloramines, and H_2_O_2_/Ag^+^) [47]—in hospital hot-water distribution systems to control microbial contamination and biofilm formation are well-documented, these treatments are not applicable in SBSs for the following reasons: the thermostatic valve does not permit the temperatures required for such treatments (>50 °C) and some plastic materials cannot support high temperatures. Moreover, despite the fact that the HCFs hot-water distribution systems supply water treated by H_2_O_2_/Ag^+^ to SBSs, the concentrations found at the SBS outlets (about 10–20 mg/L) were not able to control *Legionella* growth [41]. Therefore, the low water consumption and infrequent flushing reduced the disinfectant residuals at distal outlets, preventing adequate contact time for bacterial inactivation.

To avoid the stagnation of water and biofilm formation, the only physical treatment adopted in SBSs during the study consisted of increased flushing of water once a week for 2–3 minutes by HCF maintainers which, unfortunately, was not enough to control bacterial contamination.

During our study, the role of temperature on controlling bacterial growth was demonstrated in three SBSs of two HCFs (A and F) where, during our environmental monitoring, temperatures over 50 °C were recorded. This was compared with data recorded in these three SBSs in the presence of temperatures under 50 °C, resulting in the relative absence and lessened detection limit of *P*. *aeruginosa* and *Legionella*, respectively, under high-temperature conditions.

In these three SBSs, we found the boiler output hot water at a temperature value of around 70 °C, which consequently produced higher SBS temperatures. This transient high-temperature control on bacterial load was discussed with the SBS manufacturer, but was not implemented for a long time (for reasons previously reported).

A new protocol regarding SBS maintenance and the disinfection of components was introduced in the HCFs involved in the study. This protocol consisted of changing the thermostatic valves during functional checking by manufacturers (once per year), descaling and disinfection by immersion of showerheads in a home-made solution of sodium hypochlorite (0.5% v/v; weekly), cleaning of all surfaces and showerheads between each patient, bathtub flushing with and without showerheads (once a week), and substituting all broken bathtubs and showerheads to avoid biofilm accumulation. This protocol is still in adoption.

Unfortunately, it was not possible to associate the two cases of LD which occurred during our study with the SBS devices. This gap was due to the lack of clinical *Legionella* isolation and culture, such that matching between clinical and environmental isolates was impossible. In our experience, the lack of an epidemiological link is often associated with insufficient knowledge about the correct procedures to carry out during autopsies, which generally occurs due to a lack of sterile equipment, the use of non-sterile water, and inadequate conservation of autoptic fragments. On the other hand, the failure of isolation and culturing of clinical materials culture often occurs as a result of the initiation of broad-spectrum antibiotic treatment before a correct diagnosis [23].

The absence of literature and knowledge about SBS environments, as well as their absence in water safety plans, does not permit the comparison of our results with other experiences but, at some times, permits us to highlight the role of promoting and extending SBS surveillance programs in other facilities.

## Conclusions

SBS contamination can be minimized by implementing technical changes determined through better knowledge about SBS devices. This study supports the importance of microbiological environmental monitoring in such activities and devices still unknown, where the technologies used often pose risks of promoting infection. Therefore, we suggest the introduction of SBSs in risk assessment plans, monitoring programs, and training programs concerning the prevention of nosocomial and occupational infections in HCFs. Our future goals will involve the implementation of surveillance programs in other HCFs, as well as the involvement of local and national health authorities to support the development of guidelines for the mandatory surveillance of these devices.
